# Gender differences in symptom structure of adolescent problematic internet use: A network analysis

**DOI:** 10.1186/s13034-023-00590-2

**Published:** 2023-04-07

**Authors:** Sihan Liu, Di Zhang, Yuxin Tian, Boya Xu, Xinchun Wu

**Affiliations:** 1 Beijing Key Laboratory of Applied Experimental Psychology, Faculty of Psychology, National Demonstration Center for Experimental Psychology Education (Beijing Normal University), Beijing Normal University, No.19, Xinjiekouwai St, Haidian District, Beijing, China; 2 School of Applied Psychology, Beijing Normal University at Zhuhai, Zhuhai, Guangdong China; 3 Education and Counseling Center of Psychological Health, Ocean University of China, Qingdao, Shandong China; 4 School of Psychology, Nanjing Normal University, Nanjing, Jiangsu China

**Keywords:** Problematic Internet use, Internet addition, Gender difference, Adolescence, Network analysis, Network comparison, Symptom structure

## Abstract

**Background:**

Gender differences in problematic Internet use (PIU) have long been discussed. However, whether and how female and male adolescents differ in central symptoms and symptom associations are not fully understood.

**Methods:**

As a national survey in the Chinese mainland, 4884 adolescents (51.6% females; *M*_age_ = 13.83 ± 2.41) participated in the present study. This study applies network analysis to identify central symptoms of PIU networks in female and male adolescents and compares whether and how global and local connectivity of PIU networks differ by gender.

**Results:**

Female and male network structures of PIU were different and global strength was stronger in males than females, indicating a higher risk of chronicity of PIU among male adolescents. Specifically, “Reluctant to turn off Internet” exerted the largest effect on both genders. “Increase time online to achieve satisfaction” and “Feel depressed once not online for a while” were particularly critical to female and male adolescents, respectively. Moreover, females scored higher centralities in social withdrawal symptoms and males did so in interpersonal conflicts owing to PIU.

**Conclusions:**

These findings provide novel insights into gender differential risks and features of adolescent PIU. Differences in the core symptoms of PIU suggest that gender-specific interventions focusing on core symptoms might effectively relieve PIU and maximize treatment effects.

**Supplementary Information:**

The online version contains supplementary material available at 10.1186/s13034-023-00590-2.

## Background

The Internet has become an indispensable part of people’s daily lives, especially for adolescents who have been dubbed the “digital generation” or “digital natives” [1]. The Internet has emerged as an essential platform for offering a wide range of information and facilitating communication. However, it also brings potential problems, most notably the phenomenon of problematic Internet use (PIU, also named Internet addiction). According to China Internet Network Information Center, 19.5% of Chinese adolescents reported PIU [2]. PIU is defined as the inability to control Internet use [3] and is characterized by compulsive use, withdrawal symptoms, tolerance symptoms, interpersonal and health-related problems, and time-management problems [4]. Compared with adults, PIU may exert severer impacts on adolescents who are immature in biological, cognitive, and behavioral areas [5]. PIU is related to depressive symptoms, substance use, interpersonal impairment, and academic failures among adolescents [6, 7]. Considering the adverse consequences of PIU, how to improve the effectiveness of interventions for PIU symptoms is an important research question warranting exploration [8]. Hence, in the present study, we focused on the symptom network of PIU to inform intervention efforts, for the central symptoms in the network should be prioritized in theoretical models and serve as key treatment targets [9].

Regarding the influential factors in PIU, gender has been extensively examined as a key one [10]. Gender-related differences—including motivations, specific patterns, and types of Internet use—have been widely documented [11–13]. Most findings have identified a greater risk among males than females, such that adolescent males had a twofold higher risk of developing PIU than females, according to a meta-analysis [14]. Moreover, males reported higher degrees of PIU than females did [15]. Gender differences in PIU severity may be, at least partially, attributable to differences in specific symptoms: different symptoms may trigger differential levels of mental disorders [16]. Thus, to better understand the gender differences in PIU, it is necessary to clarify how PIU develops at the symptom level. Indeed, researchers found that adolescent males reported a higher level of obsession (e.g., feeling depressed or moody), loss of control (e.g., unable to reduce Internet use), and concealment (e.g., concealing the time spent online) than females [17]. Similarly, symptoms of depression and loneliness, diminished impulse control, and distraction were also found to be higher for males [18]. As for female adolescents, they scored higher for both social and emotional impairment than males; whereas no significant difference was evident in impulsive use between genders [19]. The above research on symptom clusters has supported that gender disparity exists in PIU symptom expressions—some symptoms are more likely to be related to a specific gender.

Nevertheless, previous investigations have several limitations. First, research on gender differences in PIU has been largely based on latent variable model [20]. Such a latent model assumes that a mental disorder is a latent variable that causes its symptoms, which may obscure the associations among symptoms [21, 22]. To overcome these shortcomings, network theorists propose that disorders composed of heterogeneous symptoms should be conceptualized as systems of causally connected symptoms [20]. Using network analysis, we could investigate gender differences in the central symptoms and symptom associations of PIU; network approach could provide a more nuanced picture of gender differences in PIU [23]. In addition, a network with stronger edges (i.e., higher global strength) may feature stronger feedback loops among symptoms and thus link to higher vulnerability to maintain a disorder [24]. Therefore, higher prevalence of PIU in male adolescents may be suggestive of higher global strength for the males’ PIU network.

Second, current knowledge of gender differences in PIU is limited to the severity of symptom levels [17–19], leaving the gender difference in PIU symptom structure largely unknown. Using network comparison, we can compare female and male networks through global (global strength: weighted absolute sum of all edges; network structure: how the nodes were connected overall) and local (edge strength: the strength of the associations between nodes; node centrality: the level of centrality) indicators [25]. From a network perspective, some novel attributes of PIU symptoms can be explored. The first one is to determine which symptoms are strongly interconnected with other symptoms (i.e., central symptoms); the second one is to investigate how they are intercorrelated (i.e., symptom associations) [9, 20]. Identifying central symptoms and symptom associations by gender can help identify essential differences in PIU between female and male adolescents. Further, it could help design more efficient, gender-specific treatments: targeting central symptoms could maximize the effects of interventions and prevent disorders from exacerbating [24]. Accordingly, network analysis focusing on symptom structure could shed new light on gender differences in adolescent PIU.

Considering all the above, this study applies network analysis to explore whether and how PIU symptom structure differs between female and male adolescents. Specifically, there are two objectives: (1) identify central symptoms of PIU in female and male adolescents; (2) compare symptom networks of PIU between genders, indicated by global and local connectivity [25]. Following the network theory that the maintenance of a disorder is based on higher global strength [24], we hypothesized that global strength would be higher in the male network.

## Methods

### Participants

The present study is part of a national project in mainland China. This project covered thirty-seven primary and secondary schools across six different regions of the Chinese mainland. The regions included the regions of North China, Central China, South China, East China, Northwest China, and Southwest China, accounting for 25.5%, 16.9%, 15.0%, 24.1%, 7.4%, and 11.0% of the total participants, respectively. The invited schools were in the average socio-economic levels of each region. To ensure that our results were based on valid responses, we embedded two response quality items that require the participants to choose the “forced” answer in the survey and used them to exclude inattentive respondents, resulting in an effective recovery of 87.5%. The final sample included 4884 adolescents: 2519 females (51.6%) and 2365 males (48.4%). Their ages ranged from 10 to 19 years (*M*_age_ = 13.83, *SD* = 2.41). Approximately half of the participants (50.4%) were the only child in the family and nearly all the adolescents (96.5%) lived with their parents.

### Procedure

We collected data through an e-questionnaire website (Questionnaire Star) from April 23 to May 7, 2020. First, we obtained permission from the participating schools for data collection. Second, we sent the website link of the e-questionnaire to teachers at the schools; we explained the purpose of our study and the ethical principles of scientific research. Finally, the teachers sent the link to students and their parents after obtaining their informed consent. We included our email address (in case further support or assistance was needed by the participating families) and an informed consent statement on the first page of the e-questionnaire. All the participants and their parents were informed about the purpose of the study, and they were free to withdraw from the study at any time. The Research Ethics Committee of Beijing Normal University approved the present study.

### Measures

We assessed PIU symptoms using the 26-item Chinese Internet Addiction Scale-Revised which has achieved high split-half reliability, test-retest reliability, and convergent validity and are widely used in Chinese adolescents [4, 22]. The scale comprises five symptom clusters: compulsive use, withdrawal symptoms, tolerance symptoms, interpersonal and health problems, and time-management problems. The detailed descriptions of PIU symptoms appear in Table 1. The responses were made on a 4-point Likert-type scale, ranging from 1 (“not like me at all”) to 4 (“completely like me”). Higher scores indicated higher levels of PIU. The internal consistency coefficient of CIAS-R was 0.97.

**Table 1:** Network Nodes of PIU Symptoms

	Symptom cluster	PIU symptom	Abbreviation
PIU11	Compulsive	Hard to control my behavior to use Internet	hard to control
PIU14	Compulsive	The first thought is Internet when I wake up	Internet first
PIU19	Compulsive	Uncontrollable to check online	uncontrollable checking
PIU20	Compulsive	Life is boring and empty without Internet	empty life
PIU22	Compulsive	Failure to cut back Internet use	failure to stop
PIU2	Withdrawal	Feel uncomfortable when not online for a while	feeling uncomfortable
PIU4	Withdrawal	Feel restless for network outages	feeling restless
PIU5	Withdrawal	Feel excited when online however tired	feeling excited
PIU10	Withdrawal	Feel depressed once not online for a while	feeling depressed
PIU16	Withdrawal	Feel like missing something once not online	feeling of missing
PIU3	Tolerance	Stay online longer than originally intended	longer than intended
PIU6	Tolerance	Reluctant to turn off Internet	reluctant to stop
PIU9	Tolerance	Spend longer time online than before	longer than before
PIU24	Tolerance	Increase time online to achieve satisfaction	increasing time for satisfaction
PIU7	Interpersonal and health	Impaired relationships with family members and peers due to Internet use	impaired relationship
PIU12	Interpersonal and health	Reduce involvement with friends in real life due to engagement in Internet	reduced involvement with friends
PIU13	Interpersonal and health	Aches in waist and back due to long-time Internet use	backache
PIU15	Interpersonal and health	Decline in academic performance due to Internet use	decline in academia
PIU17	Interpersonal and health	Fewer interactions with family members due to Internet use	fewer interactions with families
PIU18	Interpersonal and health	Fewer leisure activities in real life due to Internet use	fewer leisure activities
PIU21	Interpersonal and health	Physical deterioration due to Internet use	physical deterioration
PIU1	Time management	Complaints by other people about the large amount of time online	too much time
PIU8	Time management	Sleep less than four hours due to Internet use	less than four-hour sleep
PIU23	Time management	Less sleep for more time online	less sleep
PIU25	Time management	Eat erratically due to Internet use	erratic eating
PIU26	Time management	Sit up online and feel fatigued	sitting up online

### Data analysis

We used SPSS version 23.0 and R version 3.6.3 in RStudio 1.2.5033 for data analysis: the former for descriptive analysis and the latter for network analysis. The option setting of the e-questionnaire did not allow participants to skip any items. Thus, no item-level data were missing. We applied the *qgraph* package in R to estimate network structure. We employed a Gaussian graphical model [26], using the graphical lasso in combination with an extended Bayesian information criterion [27]. Each node represented a symptom, and the edge connected different nodes; higher thickness of the edge represented a stronger connection between nodes [20].

To identify central symptoms that were highly connected with other symptoms, we used the *centralityPlot* function to estimate the centrality of each PIU symptom, including three indicators [28]. Strength centrality represents a greater influence on the individual owing to the coexistence of several symptoms [29]; closeness centrality represents how a symptom quickly spreads to other symptoms [30]; betweenness centrality represents possible interventions when examining comorbidity, which acts as a bridge connecting the whole network [31].

We employed the *NetworkComparisonTest* function to estimate the differences between female and male networks, indicated by global and local differences. The network structure invariance test (comparing how the nodes were connected) and the global strength invariance test (comparing the weighted absolute sum of all edges) quantified global differences; the edge and node invariance tests (examining which specific edges and nodes were different) quantified local differences [25].

We applied the *bootnet* package to investigate the accuracy and stability of the networks. We examined edge-weight accuracy using the 95% confidence intervals (CIs) of the edge-weight bootstrap: higher degrees of overlapping between the CIs indicated better accuracy. Centrality stability was investigated using a case-dropping bootstrap, adopting a centrality stability coefficient as a reference index. A value higher than 0.25 is acceptable; a value higher than 0.5 is excellent [32]. For example, the value 0.5 suggests that if our sample decreases from 95 to 25% of the original sample, the correlation estimates between the subsample and the original entire sample remained 0.5.

## Results

### Network and centrality estimation in female and male adolescents

**Fig. 1:**
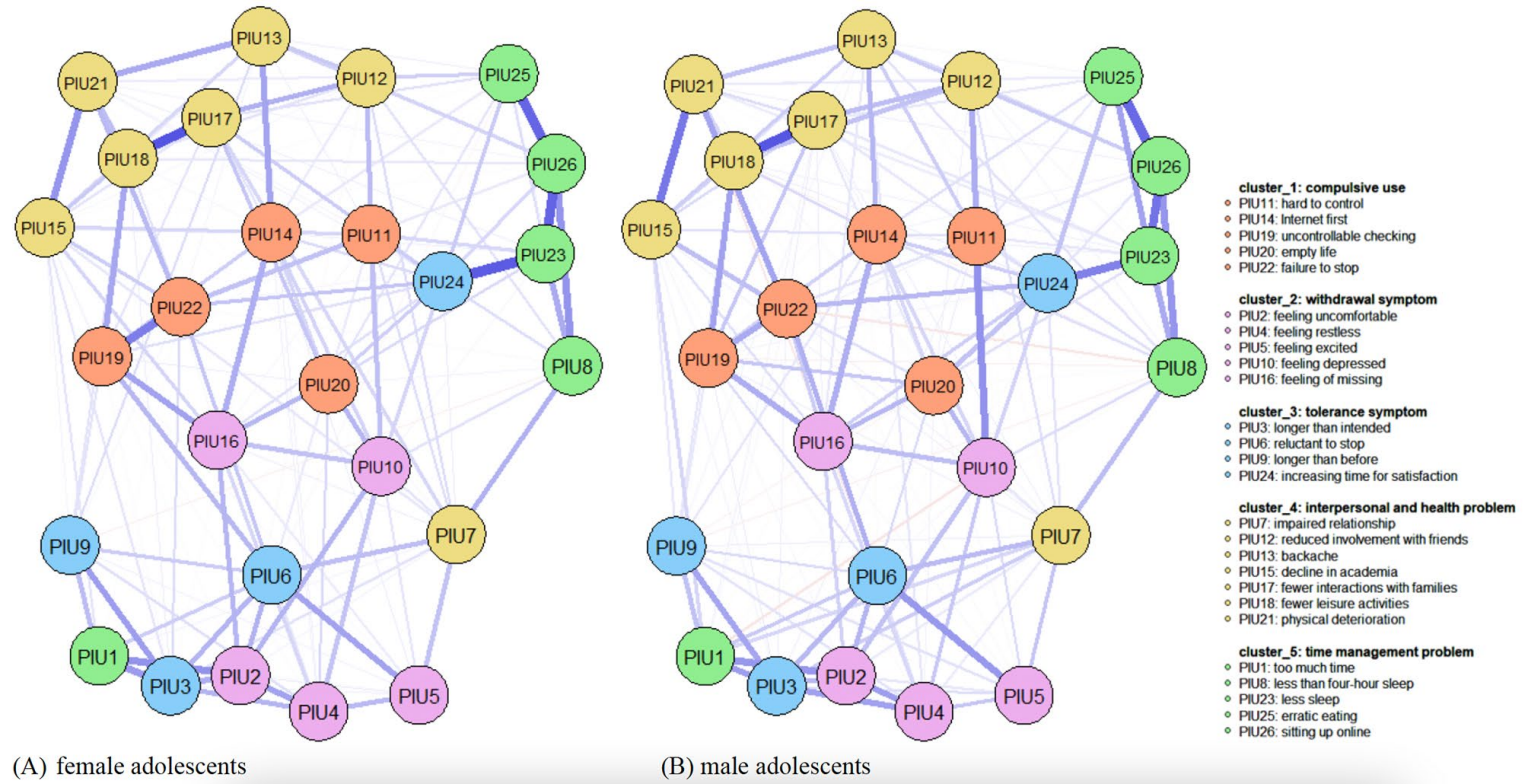
Networks of PIU symptoms in (A) female (n = 2519) and (B) male adolescents (n = 2365). Nodes represent PIU symptoms and edges represent partial correlations between symptoms. Edge thickness indicates the strength of the partial correlations (minimum and maximum edge values are set to be equal across networks) and edge color indicates the correlation valence (blue = positive; red = negative). Symptoms in same symptom clusters are shown in the same color (salmon = Compulsive Use; plum = Withdrawal; skyblue = Tolerance; golden = Interpersonal and Health; green = Time Management). See Table 1 for detail descriptions of PIU items

Networks of PIU symptoms for females and males appear in Fig. 1. There were 26 nodes and 325 edges (26*(26–1)/2) in both networks. Of the edges, 53.5% and 58.2% were non-zero-weight in female and male networks, respectively, indicating that most PIU symptoms are closely intercorrelated with each other with partial correlation estimation.

**Fig. 2:**
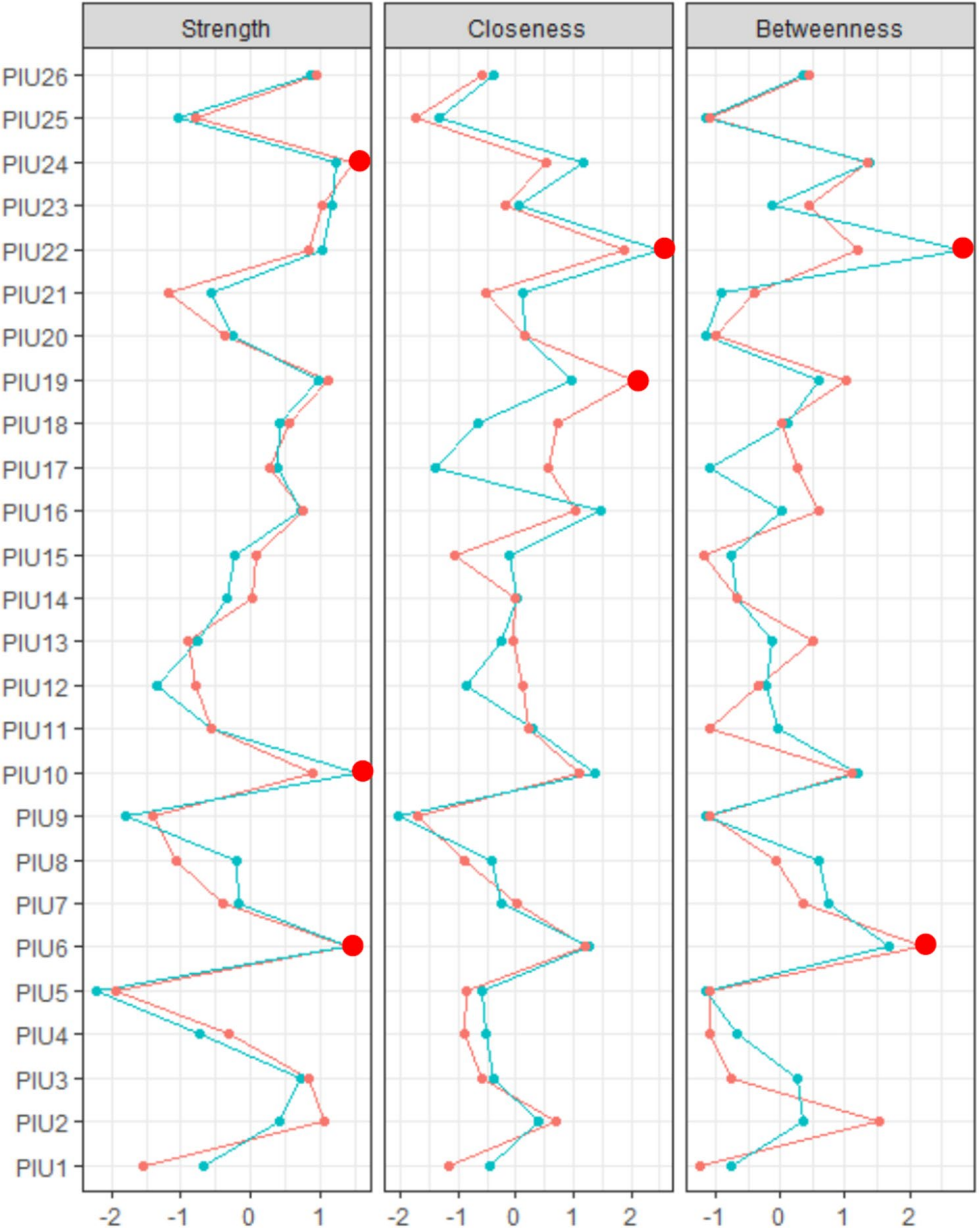
Standardized estimates of centrality for PIU symptoms by gender. The red line represents female adolescents and the blue line represents male adolescents. Red (large) dots denote the most central symptoms. See Table 1 for PIU items corresponding to each node

Figure 2 shows the centrality indexes for females and males. The red dots represent the strongest indexes. In both networks, the highest strength centrality was *reluctant to stop* (PIU6). For the female network, the highest strength centrality was “increasing time for satisfaction” (PIU24); the highest closeness centrality was “uncontrollable checking” (PIU19); and the highest betweenness centrality was “reluctant to stop” (PIU6). For the male network, the highest strength centrality was “feeling depressed” (PIU10); the highest closeness and betweenness centrality was “failure to stop” (PIU22).

The female and male networks were moderately accurate (Fig. A1): there was considerable overlap between the 95% CIs of the edge weights. The stability of both networks was excellent (Fig. A2): the female network’ strength was 0.75, closeness 0.75, and betweenness 0.60; the male’s strength was 0.75, closeness 0.60, and betweenness 0.52.

### Gender differences in global and local connectivity of networks

The global strength invariance test showed that the network strength was significantly stronger in males (global strength = 13.04) than in females (global strength = 12.40, *p* = .046). The network invariance test indicated significant differences in the network structures between genders (*p* = .048).

Table A1 presents the results of the edge invariance test. The edge invariance test revealed two notable points. First, the edge between “uncontrollable checking” (PIU19) and “failure to stop” (PIU22) was stronger in females (*p* = .019); conversely, the edges between “uncontrollable checking” (PIU19) and “empty life” (PIU20) (*p* = .026) as well as between “empty life” (PIU20) and “failure to stop” (PIU22) were stronger in males (*p* = .009). Second, “fewer interactions with families” (PIU17) was more strongly connected with “backache” (PIU13) (*p* = .007) and “physical deterioration” (PIU21) (*p* = .025) in males, whereas “fewer leisure activities” (PIU18) was more strongly connected with “sitting up online” (PIU26) (*p* = .033) and “longer than intended” (PIU3) (*p* = .019) in females.

Table A2 presents the results of the node invariance test. The node invariance test revealed significant differences in the centralities. The strength centralities of “too much time” (PIU1), “impaired relationships” (PIU7), and “backache” (PIU13) were significantly higher for males than for females (*p* = .004; *p* = .036; *p* = .048). The closeness centralities of “reduced involvement with friends” (PIU12), “fewer interactions with families” (PIU17), “fewer leisure activities” (PIU18), and “uncontrollable checking” (PIU19) were significantly higher in females than in males (*p* = .037; *p* < .001; *p* = .002; *p* = .010). The betweenness centrality of “fewer interactions with families” (PIU17) was significantly higher in females than in males (*p* = .006).

## Discussion

This study applied a novel statistical approach of network analysis to examine PIU symptomatology by gender. This study identified gender differences in global and local connectivity for adolescent PIU symptoms. The PIU symptom network of male adolescents was more densely connected than that of females, and the network structure differed between genders. “Increasing time for satisfaction” (PIU24) was a particularly central symptom in female adolescents, whereas “feeling depressed” (PIU10) was a particularly central symptom in male adolescents. In addition, female adolescents showed greater symptoms of social withdrawal than males, including “reduced involvement with friends” (PIU12) and “fewer interactions with families” (PIU17), and “fewer leisure activities” (PIU18). Conversely, male adolescents presented more interpersonal conflicts than females, including “impaired relationships” (PIU7) and “complaints by others about too much time online” (PIU1). The above findings enhance our understanding of gender differential risk and features of adolescent PIU symptoms, and also underline the need to consider gender in research and clinical practices. Our discussion focuses on the strength of centrality, the most stable feature, as found in other studies [33].

### Central symptoms of PIU in female and male adolescents

The symptoms with the highest strength centrality were either the same or different in female and male adolescents. “Reluctant to stop” (PIU6) exerted the largest effect in both genders, indicating a cross-sample central symptom. Although the participants just wanted to surf the Internet for a little while, they felt reluctant to discontinue, which suggests increasing over-dependence [4]. One reason could be that the Internet has emerged as a vital platform to fulfill social needs for adolescents, and adolescence is a critical period for searching for relatedness [34]. Another possibility is that adolescents nowadays rely greatly on the Internet for recreation. Therefore, they may experience negative feelings, such as restlessness and loneliness when disconnected from the Internet [1]. This symptom could have a severe impact on adolescents because it could allow other symptoms to occur [29].

Some core symptoms were differentiated by gender. “Increasing time for satisfaction” (PIU24) was a particularly central symptom in female adolescents, whereas “feeling depressed” (PIU10) was a particularly central symptom in male adolescents. The differential symptoms by gender may reflect different motivations and types of Internet use between female and male adolescents. One meta-analysis found that females were more likely to exhibit social media addiction and less likely to display Internet gaming disorder than males [35]. Since female adolescents often value relationships more than males, they tend to use the Internet for social networking in response to feelings of emptiness when their social needs are not otherwise fully met [36]. Once the threshold of Internet use increases, individuals require more time to reach the degrees of satisfaction previously achieved [37]. In this case, “increasing time for satisfaction” (PIU24) may greatly trigger other symptoms in females, such as “less sleep” (PIU23) found in the present study. The excessive time on Internet use may reduce adolescents’ sleep time, and this phenomenon is more common among females [38]. Thus, the central symptom of “increasing time for satisfaction” (PIU24) is more likely to lead to sleep problems in female adolescents.

In addition, we found that male adolescents feel depressed when not online, which is consistent with previous studies that depressive feeling was dominant to males’ PIU [17, 18]. One explanation is that male adolescents are more likely to engage in PIU to gain feelings of success and achievement that can be provided by online gaming [39]. While Internet use decreases or is discontinued, males may be prone to depressive feelings. Consequently, “feeling depressed” (PIU10) could severely influence other symptoms of PIU among adolescent males.

### Gender differences in global connectivity of PIU Networks

Our results support the hypothesis that global connectivity would be stronger among adolescent males than among females, which is in line with previous findings about males being at a higher risk of PIU [10, 14]. A highly connected network is more likely to develop strong self-reinforcing loops of symptom interactions [24]. Our results indicate that once male adolescents begin to engage in PIU, they tend to display a more strongly connected symptom network than females, and ultimately, males might be prone to involve in long-term PIU. One possible explanation is that males easily become addicted to Internet gaming, which impairs executive control [13]. Worse, decreased impulse control can dramatically exacerbate PIU [40]. Another explanation could be that compared with females, males tend to suppress or avoid emotions and apply fewer adaptive emotional regulation strategies [41]. Therefore, males are more likely to use the Internet as a coping strategy—although it may trigger vicious circles of negative emotion and PIU, in turn aggravating PIU symptoms. In summary, our findings help explain that chronically higher levels of PIU are more commonly observed in males than in females [42].

In addition to global strength, gender differences also exist in the network structure of PIU, which reflects different symptom associations by gender. In general, our findings indicate significant gender differences in the global connectivity of PIU networks. Below, we discuss more detailed results from the perspective of local connectivity.

### Gender differences in local connectivity of PIU Networks

We observed that local connectivity (indicated by symptom associations and central symptoms) varied by gender, which extends the literature on gender differences in PIU symptomatology. We found that female and male adolescents differed in the interacting ways and the importance levels of PIU symptoms. The following discussion focuses on two of the gender differential features of PIU symptoms: direct and indirect associations, and different effects of interpersonal symptoms as well.

First, the edge between “uncontrollable checking” (PIU19) and “failure to stop” (PIU22) differed by gender. If female adolescents are unable to resist going online, they are unable to reduce Internet use. Alternatively, if they fail to cut down their Internet use, they may feel that they are losing control. Among males, this edge existed through an irrational belief that “life is boring and empty without the Internet” [21], which is a core belief among individuals who have difficulty controlling their Internet use. Because they believe that life is boring without the Internet, they become loss of control and fail to leave it. Hence, the treatment for changing this irrational belief could be helpful to adolescent males.

In addition, interpersonal symptoms of PIU co-occurred quite differently between female and male adolescents. Among females, “fewer leisure activities” (PIU18) was highly connected with time-related problems, such as “sitting up online” (PIU26) and “longer than intended” (PIU3). Specifically, when female adolescents spend too much time on the Internet and even through the whole night, they may have not enough time and effort to put in the real life and have few leisure activities offline. As for males, “fewer interactions with families” (PIU17) was highly connected with health problems, including “backache” (PIU13) and “physical deterioration” (PIU21). The results indicate that when male adolescents indulge in the Internet and have few interactions with families, they are inclined to select the Internet for recreation instead of sports exercises, which may be harmful to their physical health. Thus, their interpersonal and health problems because of long-time Internet use are strongly connected. These gender differences extend the findings of previous studies on gender differences in the patterns and consequences of the PIU [11, 12].

Moreover, regarding core symptoms of PIU, despite some common areas across gender, several symptoms differed between female and male adolescents, most of which were related to interpersonal and health problems owing to PIU. Gender differences in the association between PIU and interpersonal impairments have been reported previously [19]. We found more specific symptom differences that female adolescents showed greater symptoms of social withdrawal than males, including “reduced involvement with friends” (PIU12), “fewer interactions with families” (PIU17), and “fewer leisure activities” (PIU18). Conversely, male adolescents presented more interpersonal conflicts than females, including “impaired relationships” (PIU7) and “complaints by others about too much time online” (PIU1). The gender differences could be explained by the different ways females and males express conflicts [43]: when adolescents experience interpersonal problems with families or friends because of their over-dependence on the Internet, females tended to express conflict covertly (e.g., fewer or avoidant interactions) and males tend to express conflict overtly (e.g., conflictive interactions).

Specifically, the Internet creates excellent opportunities for adolescents with relational resource deficits to be socially efficacious and develop meaningful relationships online [37]. On the one hand, female adolescents whose social needs are not fully met in the real life tend to use the Internet for compensation [13]. However, such evasion could further decrease their real-life interactions and quickly activate other PIU symptoms. On the other hand, male adolescents who use the Internet as a way of avoiding interpersonal conflicts may instead aggravate conflicts with families and peers and damage real-life relationships. Thus, the conflictual relationships that develop because of PIU may trigger other PIU symptoms and severely impair male adolescents [29].

### Clinical implications

The symptoms of PIU in female and male adolescents were either the same or different, which leads to gender-specific differentiated intervention strategies. For both genders, “reluctant to stop” (PIU6) should be the target symptom in interventions. Specifically, it is necessary to help adolescents understand why they do not want to reduce the time they spend on the Internet and then provide related strategies. For example, social skills deficit theory asserts that adolescents with poor social competence may be more likely to establish relationships in the anonymous virtual world than those occurring in real life, which may result in the vulnerability of PIU [44]. Therefore, interventions focused on enhancing adolescents’ social skills are critical to decreasing their PIU symptoms [45].

In addition, it is also important to focus on “increasing time for satisfaction” (PIU24) for females and “feeling depressed” (PIU10) for males. For the symptom of “increasing time for satisfaction” (PIU24), some educational programs aiming at organizing daily activities and developing new healthy Internet use concepts and habits may be effective [46]. In terms of the symptom of “feeling depressed” (PIU10), mindfulness-based cognitive behavioral intervention can regulate an addiction-related distressed emotional state [47]. Moreover, female and male adolescents showed different features of PIU symptom clusters (e.g., interpersonal problems): social withdrawal symptoms for females and interpersonal conflict problems for males, which requires differential interventions on their motivations. Meanwhile, interpersonal and time management problems are more likely to co-occur among female adolescents, whereas interpersonal and health problems are more likely to co-occur among male adolescents. Thus, cognitive behavioral therapy focused on improving time management skills may be more beneficial to female adolescents [48] and sports intervention may be more beneficial to male adolescents [49].

### Limitations and future directions

Despite its novel findings and significant implications, this study has some limitations. First, we used self-report measures to determine participants’ PIU symptoms, which could not indicate a formal diagnosis and might be limited by self-report biases [50]. Future studies on PIU are encouraged to use structured clinical interviews and collect real-world behaviors. Second, considering the investigation’s cross-sectional design, directionality between PIU symptoms should not be causally interpreted. A future longitudinal study should identify the symptoms that exhibit the highest out-strength and may strongly influence other symptoms over time.

## Conclusions

This is the first study to elucidate gender differences in adolescent PIU network characteristics; it offers novel insights into PIU symptomatology by gender. Gender-differentiated risks of PIU were present in network structure, global strength, symptom associations, and core symptoms. The PIU network of male adolescents was more densely connected than that of females, potentially indicating higher risk of chronicity of PIU in males. The core symptoms of PIU were either the same or different in female and male adolescents, which indicates that gender-specific interventions focusing on core symptoms can effectively relieve PIU symptoms and maximize treatment effects.

## Electronic supplementary material

Below is the link to the electronic supplementary material.

Supplementary Material 1

## Data Availability

The datasets and analytical code during the current study are available from the corresponding author upon reasonable request.

## List of abbreviations

PIU: Problematic Internet use
