# Supplementary material for: Gender differences in symptom structure of adolescent problematic internet use: A network analysis
Source: Child Adolesc Psychiatry Ment Health. 2023 Apr 7;17:49. doi: 10.1186/s13034-023-00590-2 (PMC10082539; doi:10.1186/s13034-023-00590-2)
Supplement: Supplementary file 1 — Supplementary Material 1 [file 13034_2023_590_MOESM1_ESM.docx]

**Gender Differences in Symptom Structure of Adolescent Problematic Internet Use: A Network Analysis**

**Online Appendix**

Additional supporting information can be found in the online version of this article.

**Figure A1.** Edge-Weight Accuracy for PIU Symptom Network in (A) Female and (B) Male Adolescents

**Figure A2.** Centrality Stability for PIU Symptom Network in (A) Female and (B) Male Adolescents

**Table A1.** Edge Invariance Test Between Female and Male Adolescents

**Table A2.** Node Invariance Test Between Female and Male Adolescents


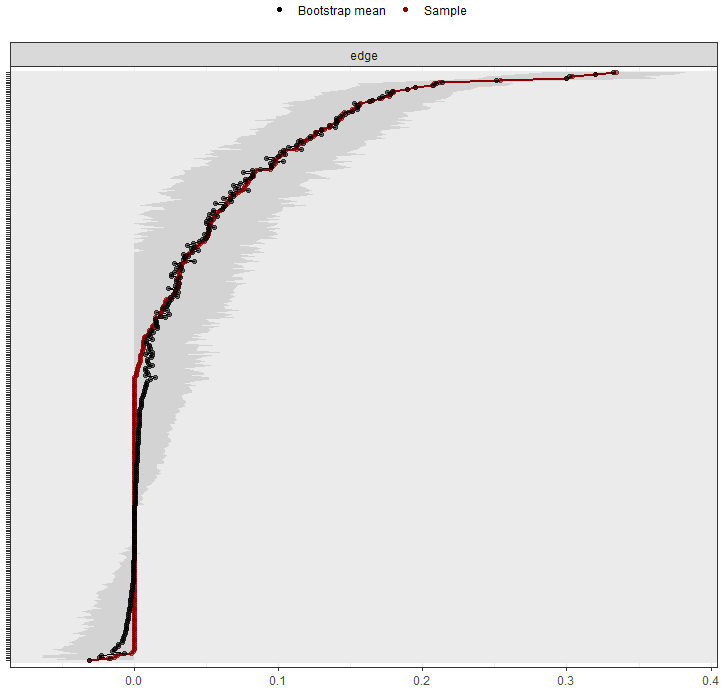


(A) female adolescents


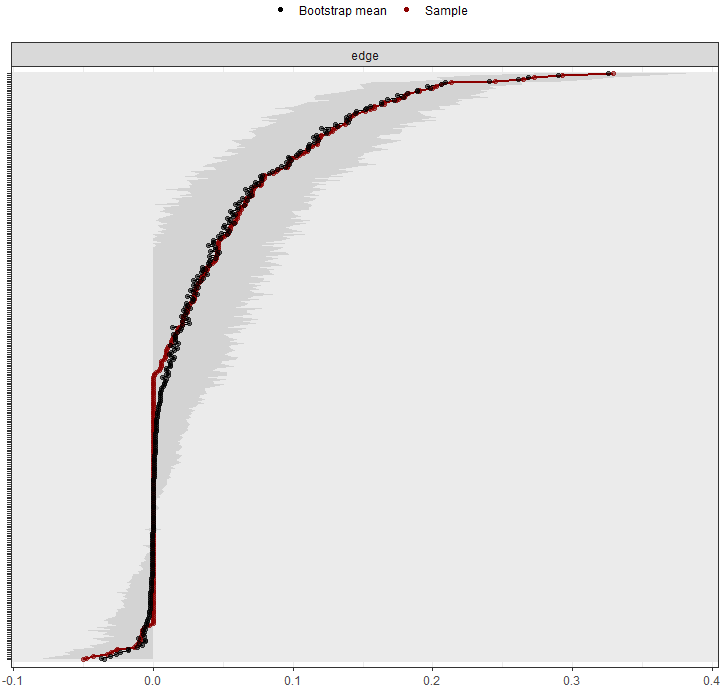


(B) male adolescents

**Appendix** **Fig. A1.** Edge-weight accuracy for PIU symptoms network in (A) female and (B) male adolescents. Bootstrapped confidence intervals of estimated edge-weights for the estimated network. The red line indicates the sample values and the gray area indicates the bootstrapped confidence intervals. Each horizontal line represents one edge of the network, ordered from the edge with the highest edge-weight to the edge with the lowest edge-weight.


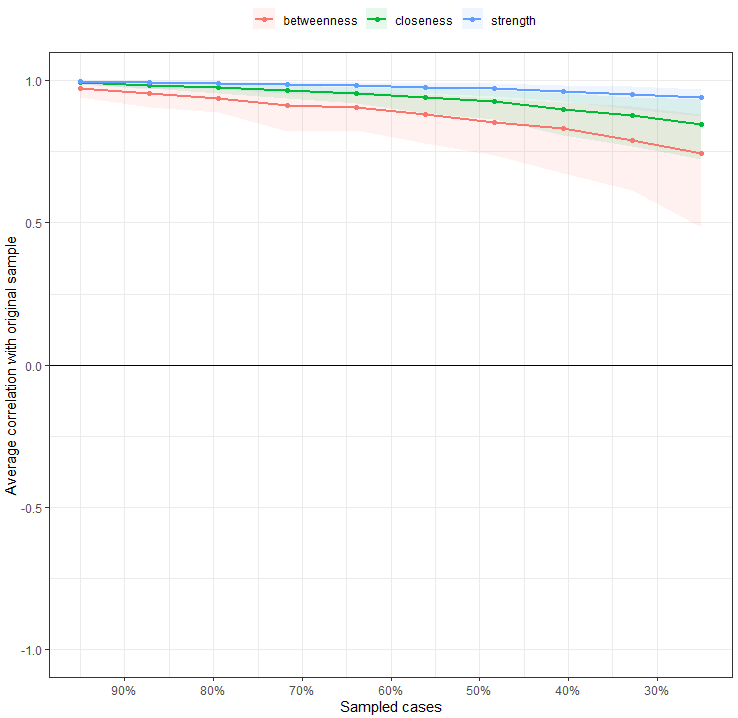


(A) female adolescents


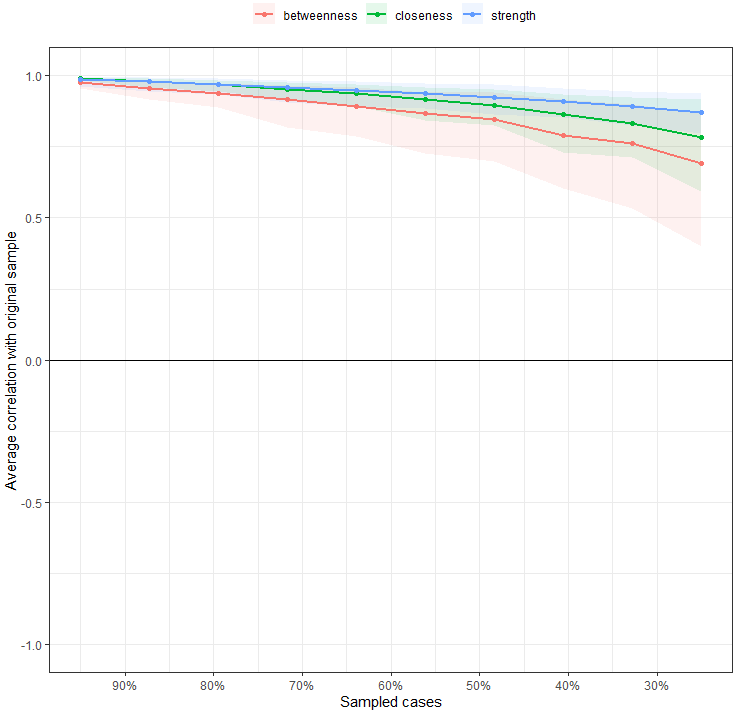


(B) male adolescents

**Appendix** **Fig. A2.** Centrality stability for PIU symptoms network in (A) female and (B) male adolescents. The x-axis illustrates the sample decrease from 95% to 25% of the original sample, and the y-axis illustrates the changes in correlation estimates between the subsample and the original entire sample. Lines indicate the means, and areas indicate the range from the 2.5th quantile to the 97.5th quantile.

**Appendix Table A1**

*Edge Invariance Test Between Female and Male Adolescents*

| edge between | | edge-weight (female) | edge-weight (male) | *p*-value |
| --- | --- | --- | --- | --- |
| PIU1 | PIU10 | 0.000 | -0.050 | 0.000 |
| PIU1 | PIU17 | 0.000 | 0.025 | 0.036 |
| PIU3 | PIU18 | 0.030 | 0.000 | 0.019 |
| PIU4 | PIU15 | 0.000 | -0.012 | 0.024 |
| PIU6 | PIU10 | 0.000 | 0.018 | 0.003 |
| PIU6 | PIU13 | 0.000 | -0.010 | 0.020 |
| PIU6 | PIU19 | 0.140 | 0.070 | 0.021 |
| PIU6 | PIU26 | 0.000 | -0.007 | 0.017 |
| PIU7 | PIU9 | 0.000 | 0.030 | 0.006 |
| PIU8 | PIU13 | 0.000 | 0.056 | 0.042 |
| PIU10 | PIU11 | 0.124 | 0.214 | 0.015 |
| PIU11 | PIU13 | 0.031 | 0.098 | 0.044 |
| PIU12 | PIU19 | 0.000 | 0.011 | 0.000 |
| PIU12 | PIU25 | 0.094 | 0.006 | 0.014 |
| PIU13 | PIU17 | 0.000 | 0.022 | 0.007 |
| PIU13 | PIU19 | 0.000 | -0.007 | 0.015 |
| PIU13 | PIU21 | 0.165 | 0.120 | 0.050 |
| PIU15 | PIU25 | 0.008 | 0.000 | 0.041 |
| PIU16 | PIU22 | 0.014 | 0.099 | 0.016 |
| PIU17 | PIU21 | 0.000 | 0.066 | 0.025 |
| PIU18 | PIU26 | 0.044 | 0.000 | 0.033 |
| PIU19 | PIU20 | 0.032 | 0.110 | 0.026 |
| PIU19 | PIU22 | 0.254 | 0.165 | 0.019 |
| PIU20 | PIU22 | 0.000 | 0.063 | 0.009 |
| PIU20 | PIU25 | 0.050 | 0.000 | 0.007 |
| PIU23 | PIU25 | 0.063 | 0.180 | 0.008 |

*Note.* Only present the results *p < .05.*

**Appendix Table A2**

*Node Invariance Test Between Female and Male Adolescents*

| Node | Strength(*p*) | Closeness(*p*) | Betweenness(*p*) |
| --- | --- | --- | --- |
| PIU1 | 0.004 | 0.517 | 0.367 |
| PIU2 | 0.395 | 0.232 | 0.263 |
| PIU3 | 0.554 | 0.776 | 0.247 |
| PIU4 | 0.955 | 0.956 | 0.647 |
| PIU5 | 0.401 | 0.831 | 0.638 |
| PIU6 | 0.801 | 0.521 | 0.726 |
| PIU7 | 0.036 | 0.287 | 0.632 |
| PIU8 | 0.152 | 0.847 | 0.384 |
| PIU9 | 0.796 | 0.270 | 0.602 |
| PIU10 | 0.072 | 0.728 | 0.910 |
| PIU11 | 0.166 | 0.598 | 0.078 |
| PIU12 | 0.851 | 0.037 | 0.895 |
| PIU13 | 0.048 | 0.306 | 0.348 |
| PIU14 | 0.988 | 0.524 | 0.873 |
| PIU15 | 0.847 | 0.374 | 0.407 |
| PIU16 | 0.376 | 0.954 | 0.588 |
| PIU17 | 0.238 | 0.000 | 0.006 |
| PIU18 | 0.729 | 0.002 | 0.916 |
| PIU19 | 0.802 | 0.010 | 0.642 |
| PIU20 | 0.316 | 0.470 | 0.579 |
| PIU21 | 0.056 | 0.681 | 0.339 |
| PIU22 | 0.397 | 0.952 | 0.106 |
| PIU23 | 0.376 | 0.734 | 0.418 |
| PIU24 | 0.894 | 0.825 | 0.978 |
| PIU25 | 0.689 | 0.855 | 0.736 |
| PIU26 | 0.719 | 0.779 | 0.963 |
